# Image guided IMRT dosimetry using anatomy specific MOSFET configurations

**DOI:** 10.1120/jacmp.v9i3.2798

**Published:** 2008-06-23

**Authors:** Md Nurul Amin, Bern Norrlinger, Robert Heaton, Mohammad Islam

**Affiliations:** ^1^ Department of Radiation Physics Princess Margaret Hospital University Health Network Toronto Ontario Canada; ^2^ Department of Radiation Oncology University of Toronto Toronto Ontario Canada

**Keywords:** IMRT quality assurance, MOSFET, Image guidance, Radiation therapy

## Abstract

We have investigated the feasibility of using a set of multiple MOSFETs in conjunction with the mobileMOSFET wireless dosimetry system, to perform a comprehensive and efficient quality assurance (QA) of IMRT plans. Anatomy specific MOSFET configurations incorporating 5 MOSFETs have been developed for a specially designed IMRT dosimetry phantom. Kilovoltage cone beam computed tomography (kV CBCT) imaging was used to increase the positional precision and accuracy of the detectors and phantom, and so minimize dosimetric uncertainties in high dose gradient regions. The effectiveness of the MOSFET based dose measurements was evaluated by comparing the corresponding doses measured by an ion chamber. For 20 head and neck IMRT plans the agreement between the MOSFET and ionization chamber dose measurements was found to be within −0.26±0.88% and 0.06±1.94% (1σ) for measurement points in the high dose and low dose respectively. A precision of 1 mm in detector positioning was achieved by using the X‐Ray Volume Imaging (XVI) kV CBCT system available with the Elekta Synergy Linear Accelerator. Using the anatomy specific MOSFET configurations, simultaneous measurements were made at five strategically located points covering high dose and low dose regions. The agreement between measurements and calculated doses by the treatment planning system for head and neck and prostate IMRT plans was found to be within 0.47±2.45%. The results indicate that a cylindrical phantom incorporating multiple MOSFET detectors arranged in an anatomy specific configuration, in conjunction with image guidance, can be utilized to perform a comprehensive and efficient quality assurance of IMRT plans.

PACS number: 87.55.Qr

## I. INTRODUCTION

Intensity modulated radiation therapy (IMRT) is rapidly becoming the standard of practice for radiation therapy of cancer patients, especially when critical normal structures are situated in the vicinity of target volumes. Due to the complexity of the planning and treatment delivery process of IMRT, it is crucial to perform dosimetric quality assurance for each patient plan.^1^, ^2^ With the increased utilization of IMRT techniques, the modern RT department is facing an enormous quality assurance workload burden for patient specific treatment plan validation. While various methods of IMRT plan validation have been described,^3^, ^7^ the most common method involves dose measurements by ionization chamber in a phantom for single or multiple points in high dose and dose avoiding regions. Additionally, to ensure the integrity of the entire radiation field, fluence maps are captured by films or electronic portal imaging devices (EPID) and compared with those calculated by the treatment planning system (TPS). Point dose measurements with ion chambers are inefficient, requiring a number of steps: (a) calculation of the dose distribution in the phantom data set by importing the IM treatment fields from the patient's IMRT plan (b) careful selection of measurement points in areas of low dose gradient (c) calculation of the mean dose for the ion chamber volume, and finally (d) setting up the phantom and ion chamber on the treatment machine and performing the measurements. The time and effort required for multiple point measurements involves repetitions of steps (b), (c) and (d). On the other hand, having simultaneous multiple ion‐chambers will perturb the dose distribution. For treatment sites such as head and neck, where many critical organs may be present around the target volume, two or more point dose measurements may be required, and therefore require substantial amount of additional time and resources. In addition, the volume averaging effect^(8)^ of an ion chamber may introduce significant uncertainties if the measurement point is not within a low dose gradient region.

We have investigated the use of multiple MOSFET (Metal‐Oxide Semiconductor Field‐Effect Transistor) sensors in conjunction with the mobileMOSFET system (Best Medical Canada, Ottawa, ON) to perform efficient and comprehensive dose measurements for IMRT plan verification. Anatomy specific MOSFET configurations corresponding to prostate and head and neck sites were developed. These configurations can be designed to be incorporated into a phantom module for the IMRT phantom, and can accommodate up to five MOSFETs at carefully selected points corresponding to planning target volume (PTV) and organs at risk (OAR). Optimum locations for the MOSFETs were determined for each anatomic site based upon the IMRT plans of ten typical patients.

The MOSFET sensitive area is 0.2 mm x 0.2 mm and therefore can be considered as a point detector. However, because of their small size, a high positional accuracy is required for these detectors. Since image guidance systems, based on the kV CBCT, are now becoming standard equipment on modern linear accelerators, we have utilized such a system to accurately position the detectors prior to dose measurements. In this report we describe our experience with anatomy specific MOSFET configurations for IMRT plan verification, positioned in the treatment field using image guidance.

## II. MATERIALS AND METHODS

This study was performed on an Elekta Synergy linear accelerator (Elekta, Stockholm, Sweden) equipped with an 80 leaf‐MLC and XVI CBCT system. IMRT plans using 6 MV beams were generated using the Pinnacle TPS (version 7.6C; Philips Medical Systems, Andover, MA).

The mobileMOSFET system (TN‐RD‐70‐W) with high bias setting and high sensitivity microMOSFETs (TN‐1002RDM) were used for this investigation. The system uses a battery powered MOSFET reader, which communicates wirelessly using Bluetooth, to a transceiver (Model: TN‐RD‐38) placed within 10 meters of the reader. This wireless transceiver is connected to a PC‐based readout system. Up to eight readers can be used simultaneously with the system, with each reader supporting up to 5 MOSFETs. The active detection area of the MOSFET is 0.04 mm^2^. The detailed structure and technical aspects and design of MOSFET detectors have been described previously by several authors.^9^, ^12^

**Figure 1:**
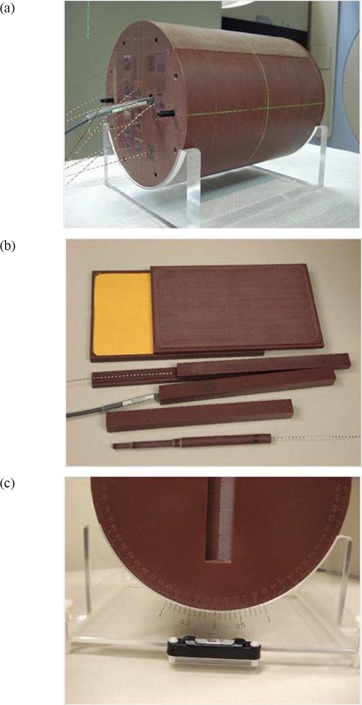
IMRT Phantom: (a) phantom with multiple MOSFETs positioned for image guided dosimetry, (b) film, MOSFET and ion chamber holder, (c) phantom end plate showing angular scale.

This study used a in‐house designed and manufactured dosimetry phantom, referred to here as the IMRT phantom. The IMRT phantom is a precisely machined cylindrical phantom, 25 cm in length and 20 cm in diameter, made of Solid Water (Gammex rmi, Middleton, WI), supported on a clear plastic base. The phantom can incorporate a number of different modules including: (a) ion chamber holders (b) MOSFET holders and (c) light‐tight film cassette. The core of the cylinder can be easily assembled with each of the modules in such a way that the user has the flexibility of placing the sensitive volume of multiple ion chambers or MOSFETs at various axial and radial positions within the cylinder. As shown in Fig. 1, both the ion chamber (0.6 cc Farmer type) and MOSFET (standard and micro) have 2×2×25 cm specialized holders which can be inserted into the phantom.

Phantom set up is facilitated by 3 lines inscribed on the surface along the length of the phantom, which can be aligned with the sagittal and lateral lasers. The phantom base (as shown in Fig. 1) can be adjusted with a spirit level to complete the phantom set up. An angular scale with 0.1° resolution is provided to permit precise angular positioning.

### A. Dosimetric characterization

The mobileMOSFET is a new system for using MOSFETs more efficiently in a clinical environment that has not been previously described in the literature. Consequently, a careful study of the dosimetry system was performed to ensure that the system characteristics and properties are consistent with other systems using the same type of detector.

#### 1. Sensitivity, linearity and reproducibility

All the measurements in this section were referenced to doses measured with a calibrated 0.6 cc Farmer type ionization chamber (NE 2571). The sensitivity of the system, in terms of mV/cGy, was determined by performing measurements with MOSFETs irradiated to a dose of 100 cGy using 6 MV X‐rays. The linearity of response as a function of dose was assessed by exposing the MOSFETs to absorbed doses in the range of 1 cGy to 700 cGy. We have investigated the reproducibility of response (short term) for doses 1 cGy to 200 cGy by performing 10 sequential measurements at each dose setting. The reproducibility is defined as the standard deviation of the mean value, expressed as a percent. All the above measurements were performed by placing the MOSFETS in a block of Solid Water (20×20×20 cm) at a depth of 1.5 cm with a field size of 10×10 cm and 98.5 cm SSD.

#### 2. Angular dependence

The angular dependence of the high sensitivity microMOSFET was investigated using the IMRT phantom. MOSFETs were placed precisely at the centre of the phantom, and the phantom was aligned to the isocenter using the machine front pointer. This measurement geometry ensures that the central axis of the radiation beam from any gantry angle traverses 10 cm of Solid Water during detector exposure, and simulates the radiation exposure conditions used in IMRT plan verification. Measurements were performed at 30° intervals for gantry angles between 0° to 330° using 50 MU each.

#### 3. Performance study in comparison with ion chamber

The performance of MOSFET detectors for IMRT dose verification was assessed through a comparison to ion chamber results for 20 head and neck step‐and‐shoot plans. The field parameters of these plans were transferred to the CT image set of the IMRT dosimetry phantom and full three dimensional dose calculations were performed. Subsequently, two points of interest were selected: a high dose point (HD) within the planning target volume (PTV) and a low dose point (LD) corresponding to an avoided organ at risk. Points were selected in low dose gradient regions and ion chamber dose‐volume averaging was accounted for in the calculation. The dose to these points were measured in phantom using both 0.6 cc Farmer‐type ionization chambers (Model: NE2571) and microMOSFETs (Model: TN‐1002RDM).

### B. Anatomy specific MOSFET configurations

**Figure 2:**
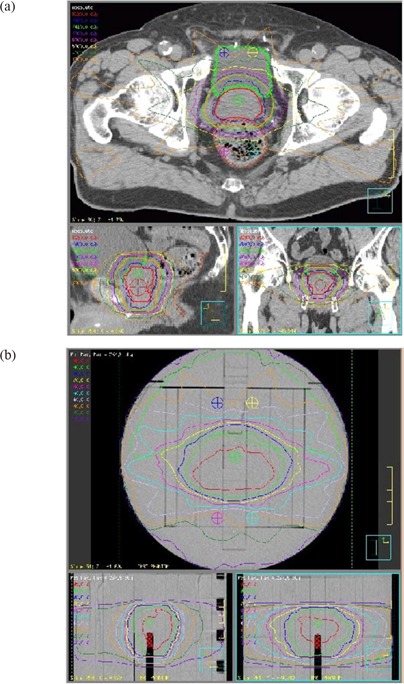
IMRT prostate plan showing MOSFET positions after plan export to the phantom (a) patient plan (b) corresponding plan on IMRT phantom.

**Figure 3:**
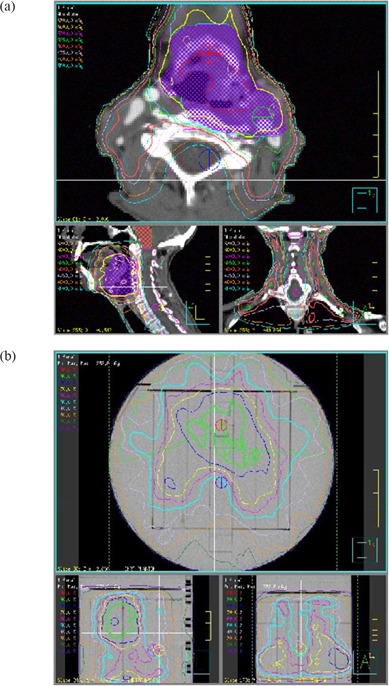
IMRT head and neck plan showing MOSFET positions after plan export to the phantom (a) patient plan (b) corresponding plan on IMRT phantom.

Anatomy specific fixed configurations of 5 MOSFETs were used to verify typical prostate and head and neck (nasopharynx and base of tongue) IMRT plans. Clinical IMRT plans for 10 prostate and 10 head and neck treatments were used to determine the average position of 5 MOSFET detectors within the phantom. To accommodate patient variations as well as the high gradients typical of IMRT plans, 2 measurement points were dedicated to sampling the PTV region for head and neck type treatments, with the remaining 3 points corresponding to the average location of the spinal cord, as shown in Fig. 3. In contrast, for prostate plans one detector was placed in a location corresponding to the approximate center of the PTV, while two detectors were placed to corresponding to the typical bladder and rectum locations as shown in Fig. 2. The coordinates of these fixed points for each site relative to the center of the IMRT phantom are listed in Table 1.

**Table 1:** Dosimeter positions, with respect to the geometric centre of the phantom, for three IMRT MOSFET configurations: prostate, nasopharynx and base of tongue.

*Site*	*ROI*	*Lat. (X) cm*	*AP/PA (Y) cm*	*Sup/Inf (Z) cm*
Prostate	PTV‐1	0.05	−24.8	−1.6
	Bladder‐1	−1.45	−19.8	−1.6
	Bladder‐2	1.55	−19.8	−1.6
	Rectum‐1	−1.45	−30.1	−1.6
	Rectum‐2	1.55	−30.1	−1.6
Nasopharynx	PTV‐1	0.05	−22.8	0
	PTV‐2	−2.45	−23.1	0
	Spinal cord‐1	0.05	−27.8	0
	Spinal cord‐1	−0.45	−29.1	0
	Spinal cord‐1	0.55	−30.1	0
Base of tongue	PTV‐1	0.05	−21.8	−2
	PTV‐2	2.55	−24.8	−2
	Spinal cord‐1	0.05	−26.8	−2
	Spinal cord‐1	−0.45	−28.8	1
	Spinal cord‐1	−0.45	−27.8	1

### C. Accurate positioning of MOSFETs with image guidance

The small 0.2×0.2 mm detection area of the MOSFET and the presence of high gradients in IMRT plans necessitated precise positioning with respect to the isocenter. Precise placement of the MOSFETs in this study was achieved using the XVI CBCT system. This system is capable of reproducibly positioning a patient to better than ±1 mm,^(13)^ while the laser system typically provides positioning within ±2 mm.

The CBCT based image guidance system allows 3D volumetric image data acquisition while the patient is on the treatment couch. The system consists of a kilovoltage (kV) X‐ray source and an aSi detector panel mounted on the treatment gantry, orthogonal to the MV beam axis. The center of the imaging system is coincident with the isocenter of the treatment system. A detailed description of kV cone beam CT as well as image guidance procedures have been reported in the literature.^14^, ^16^

### D. CT based dose calculation in phantom

**Figure 4:**
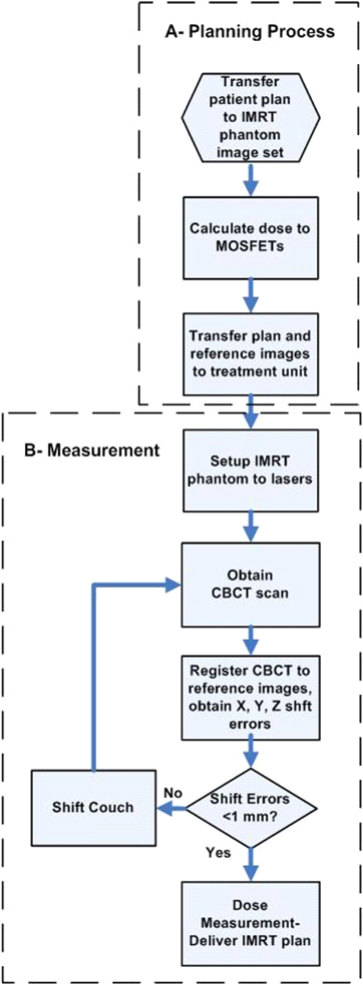
Flow chart showing the process of routine IG IMRT dosimetry in phantom (a) dose calculation (b) dose measurement.

A CT image set, consisting of 2 mm slice thickness, of the phantom containing the MOSFET detectors was acquired on a Philips Brilliance CT simulator (Philips Medical System, Netherlands) at 120 kV and 250 mAs and exported to the treatment planning system. Plan parameters were transferred to the reference image set and the dose at each MOSFET position was calculated with a dose calculation grid of 2.5 mm for each plan investigated. The phantom CT image set, along with the beam parameters, were then exported to the XVI CBCT system as a reference image set. This process is illustrated in (Fig. 4a).

### E. IMRT plan verification with anatomic specific MOSFET configuration

**Figure 5:**
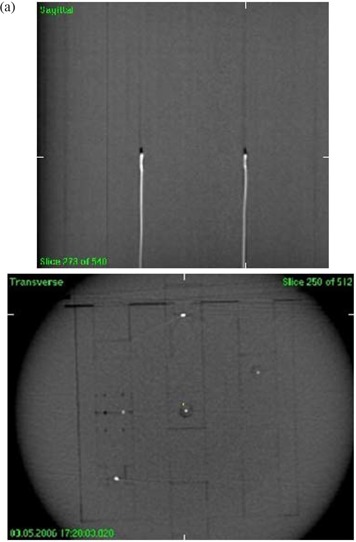
Cone beam CT images of five MOSFETs in the Solid Water phantom: (a) mid‐sagittal view and (b) cross sectional view.

The phantom was placed on a standard head and neck couch extension and aligned to the laser isocenter using inscribed marks on the phantom. The position of the phantom was determined from a 360° CBCT scan (Fig. 5) and then registered with the reference CT images. A total of 650 projections were acquired with 120 kV (1040 mAs total) to reconstruct a 1024×1024 resolution image matrix. The reconstructed CBCT image was used to register and correct for the 3D x, y, z displacements of the MOSFET detectors. This process is illustrated in (Fig. 4b).

A total of 13 new patient plans, from both prostate and head and neck sites, were utilized to verify the dosimetry of the anatomic configurations. To take into account the day‐to‐day output variation of the linear accelerator, as well as individual detector response, reference readings were obtained for all the MOSFETs using 100 cGy dose prior to performing the dose measurements of the IMRT plans.

## III. RESULTS

### A. Dosimetric characterization

#### 1. Sensitivity, linearity and reproducibility

A sensitivity of 7.75±0.02 mV/cGy was measured for a batch of 10 high sensitivity microMOSFETs under high bias settings. The MOSFET responses were found to be highly linear $\left(R^{2}=1\right)$ with dose over the range of 1 cGy to 700 cGy. The reproducibility values at the dose levels of 100 cGy, 50 cGy and 1 cGy were found to be 0.7%, 1% and 2.1%, respectively. These reproducibility results are an improvement compared to the previously reported values.^(9)^ This improvement can be attributed to the new mobileMOSFET automated readout system.

#### 2. Angular Dependence

**Figure 6:**
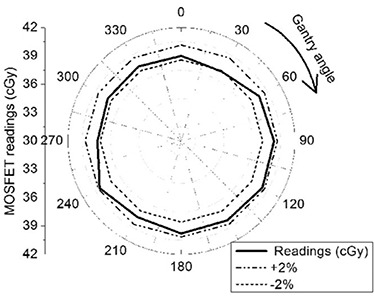
Radial plot to show the angular variation of MOSFET sensitivity from 0° to 330°.

The relative angular variation in sensitivity is shown in Fig. 6. A maximum angular sensitivity variation of approximately 2% was observed; this result is similar to the results reported earlier.(9,17–18)

#### 3. Performance study in comparison with ion chamber

**Figure 7:**
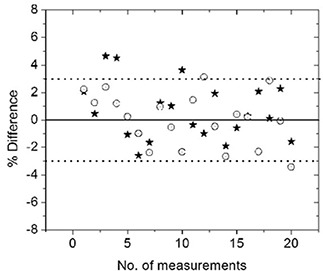
A comparison of dose measured using ionization chamber and MOSFET for 20 head and neck patients. The circles (◯) represent HD points and stars (*) represent LD points.

The dose delivered to points in the PTV and OAR were measured for 20 head and neck patients using the mobileMOSFET system and an ionization chamber. Fig. 7 shows a scatter plot of percentage dose difference between MOSFET and ionization chamber for all 20 head and neck plans. The difference in most cases is within ±3% with an average of 0.7±2.1% for the high dose point and 0.1±1.9% for the low dose point. Only a few points gave more than 3% variations. These were investigated and were found in each instance to correspond to measurement points in relatively high dose gradient regions.

### B. IMRT plan verification with anatomic specific MOSFET configuration

**Figure 8:**
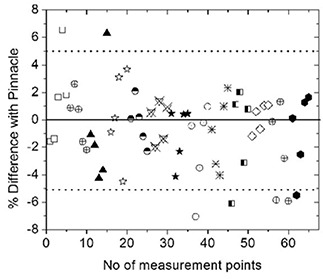
A comparison of dose measured using MOSFET and plan dose for 13 different IMRT patients. The different shapes represent different patients.

The results of 13 patient plans (5 head and neck, and 8 prostate), in terms of agreement of 5 MOSFET measured doses with those calculated by Pinnacle, are shown in Fig. 8. As shown, 72% of points agree within ±3% and 90% within 5%, while 10% of the points show more than a 5% variation. The points of higher disagreement appear to be in the high dose gradient regions and may be attributed to residual positioning as well as calculation uncertainties. However, it is noted that in all the plans tested, at least four MOSFET measured doses were within ±5% of TPS calculated values.

## IV. DISCUSSION

IMRT validation measurements at our institution are currently performed using a cylindrical phantom with a 0.6 cc ionization chamber, at 2 patient specific measurement points, corresponding to the location of the PTV and OAR. A significant amount of pre‐measurement work is put into identifying positions for these measurements that avoids high dose gradients. In spite of this effort, it is recognized that gradients in the chamber region coupled with set‐up uncertainties can contribute significantly to the measurement uncertainty. For a composite field measurement, a tolerance of ±3% has been adopted. Plans, which fall outside this tolerance, are subjected to further investigation to resolve the discrepancy.

The use of the image guided MOSFET system for these measurements avoids many of these issues, but introduces the uncertainties of MOSFET angular response and reproducibility. These properties combine to yield a 3σ dosimetric uncertainty for measurement of 4.6%, based on the measured standard deviation for reproducibility and angular dependence. The small size of the detectors greatly reduces the impact of gradients to the point where positioning uncertainties are the dominant remaining contribution; with the imaging techniques utilized in this study, any given dosimeter can be placed to within approximately 1 mm of the planned position. For points located away from large dose gradients, dosimetric changes over this distance (i.e. 1 mm) are typically on the order of 1%, as we have experienced during careful measurements of IMRT fields with an ionization chamber. Combining all these uncertainties in quadrature, the MOSFET dosimetric uncertainty yields a total uncertainty of approximately 5.0%, which has been adopted as our tolerance level for triggering a more extensive investigation into the plan dosimetry.

It should be noted that a ±5% tolerance for each measurement point does not factor in the largest possible measurement uncertainty that could occur. The maximum dose gradient, based on penumbra considerations, is approximately 10%/mm, so that higher deviations may be observed from time to time for any given point. By using multiple measurement points together with anatomy specific configuration, we found that accurate measurements in both the target and avoidance regions can be performed without resorting to patient specific point placement. While dose deviations from positional uncertainties can still occur, the use of multiple measurement points in this study ensured that at least one point in each of the target and avoidance regions provided a measurement free from large gradient effects.

## V. CONCLUSIONS

The use of the mobileMOSFET system, in conjunction with image guidance, has been investigated for high precision and comprehensive dosimetry of patient specific IMRT. The results of the investigation show that the sensitivity, accuracy and reproducibility of the high sensitivity microMOSFET operated with the mobileMOSFET system provides a reliable and practical dosimetry solution for patient specific IMRT QA.

The fixed configuration MOSFET module, embedded in a specially designed phantom, allows simultaneous multiple point dose measurements and provides efficiency in the quality assurance process. The requirement for high positional accuracy when using MOSFETs, which are point detectors, can be achieved through the use of image guidance technology. The extra time taken by the image guidance procedure is easily compensated for by the efficiency gained by performing simultaneous multiple point measurements. As another advantage, image guidance allows the user to verify the position of the MOSFETs, rather than rely on external markers on the measurement phantom.

## ACKNOWLEDGEMENTS

This work is supported in part by the Ontario Consortium for Image Guided Therapy and Surgery (OCITS). The authors would like to acknowledge the help of Yuen Wong (Machinist), for his extraordinary precision work in manufacturing the phantom modules and Mohammad Rahman for his help with the various stages of imaging the phantom.
